# Supplementary figures and images for: FMRFa receptor stimulated Ca2+ signals alter the activity of flight modulating central dopaminergic neurons in Drosophila melanogaster
Source: PLoS Genet. 2018 Aug 15;14(8):e1007459. doi: 10.1371/journal.pgen.1007459 (PMC6110513; doi:10.1371/journal.pgen.1007459)

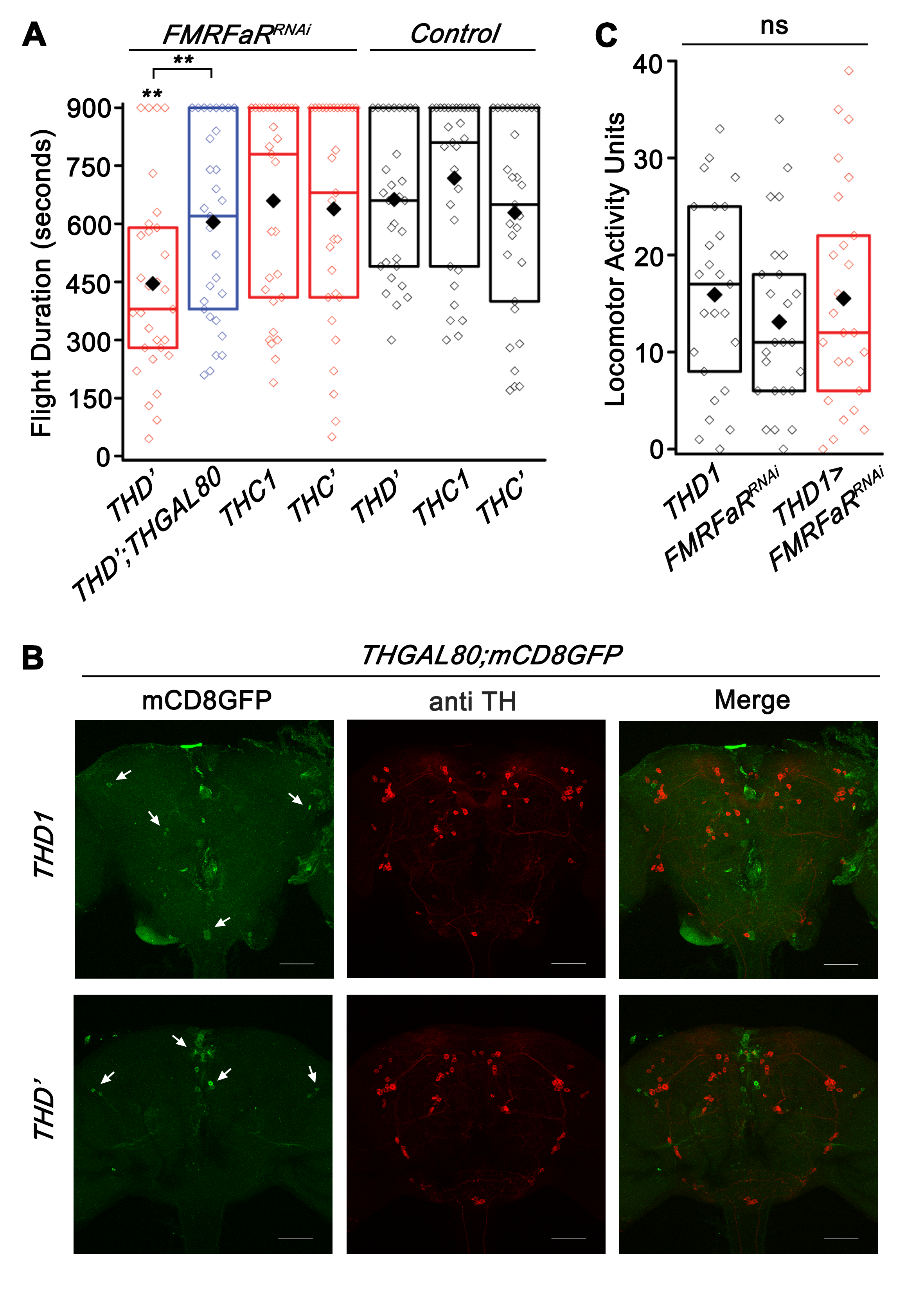

Supplement: S1 Fig — (A) Flight durations observed with knockdown of FMRFaR in a few TH subset domains, namely, THD’, THC1 and THC’ as compared to their genotypic controls (THD’/+, THC1/+, THC’/+ in black and FMRFaRRNAi/+ shown in Fig 1A; n≥30, **p<0.01, Mann-Whitney U-test). The ‘+’ in all control genotypes denotes the wild-type, Canton-S, allele. (B) Immunostaining of adult brains with TH antibody in the indicated genotypes (THD1;THGAL80>mCD8GFP, THD’;THGAL80>mCD8GFP). Neurons that were positive for GFP, but not TH are indicated by white arrows. Scale bars represent 50 μm. (C) Locomotor activity measured for control adult flies (THD1/+ and FMRFaRRNAi/+) were not different from that observed with THD1>FMRFaRRNAi flies (n = 25, p>0.05, Mann-Whitney U-test). (TIF) [file pgen.1007459.s001.tif]

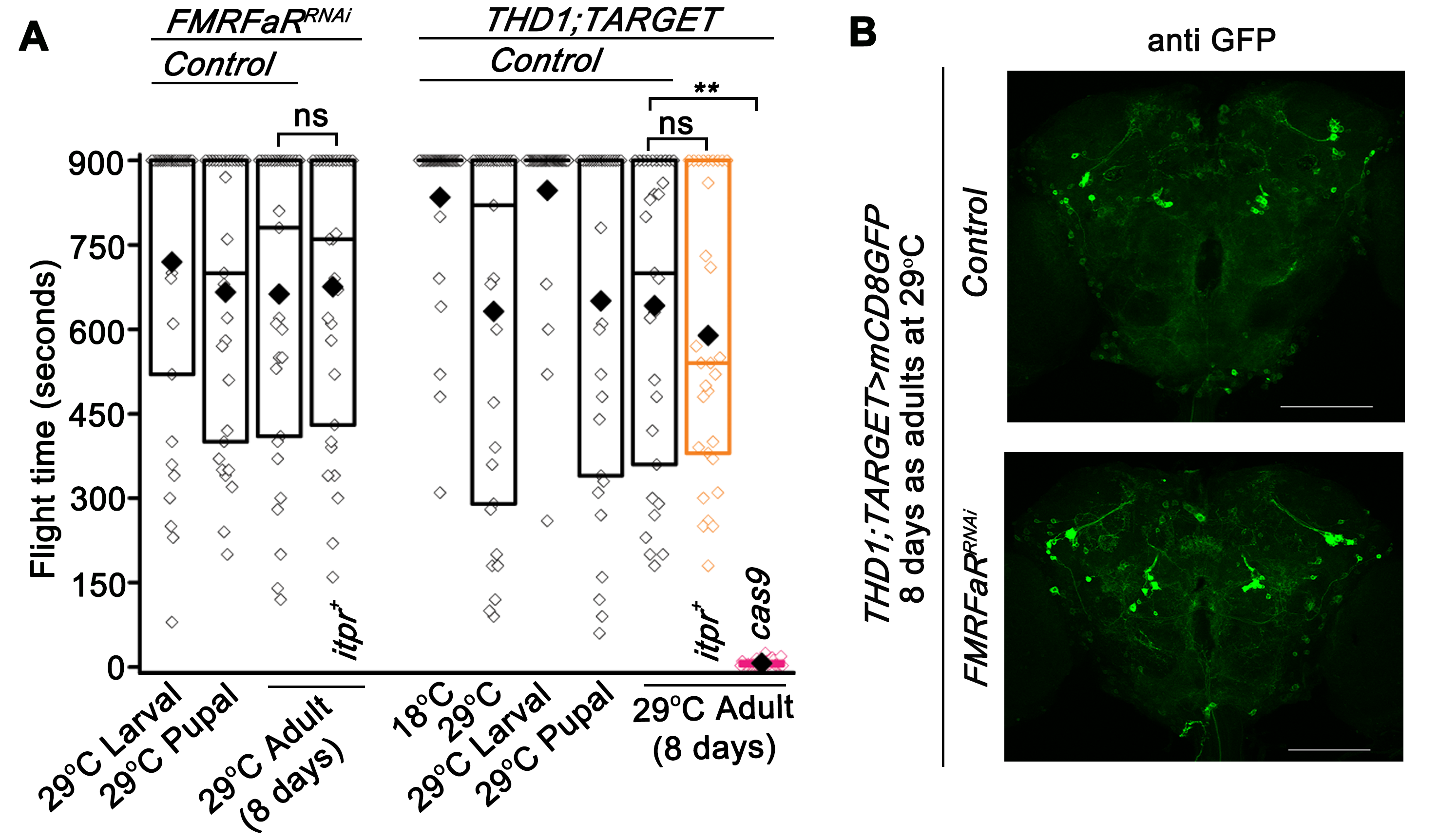

Supplement: S2 Fig — (A) Genotypic controls for the TARGET experiment (FMRFaRRNAi/+ and THD1;TubGAL80ts Control) tested for flight under various temperature shift conditions. Other controls in the graph include itpr+/+;FMRFaRRNAi/+ (4th bar from the left in black), THD1;TubGAL80ts>itpr+ (orange bar) and THD1;TubGAL80ts>cas9 (pink bar). Comparisons are shown by horizontal lines (n = 30, **p<0.01, ns–not significant; Mann-Whitney U-test). (B) Immunohistochemical staining of 8 day old adult brains using anti-GFP antibody in control (above; THD1;TubGAL80ts>mCD8GFP Control) and FMRFaR knockdown (below; THD1;TubGAL80ts>mCD8GFP;FMRFaRRNAi) conditions. Scale bars represent 100 μm. (TIF) [file pgen.1007459.s002.tif]

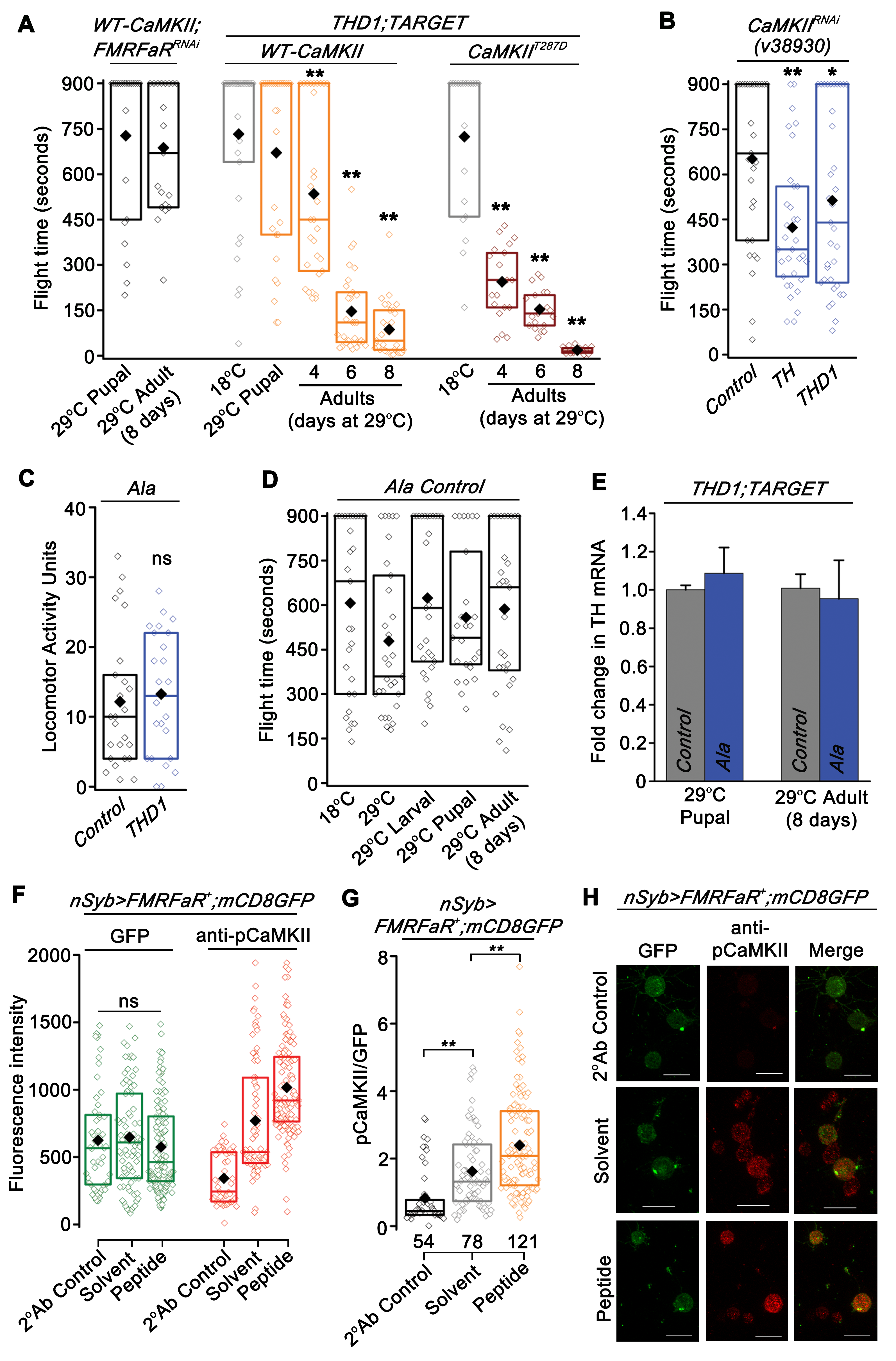

Supplement: S3 Fig — (A) Flight durations observed for a strain used in Fig 4A (WT-CaMKII/+;FMRFaRRNAi/+). Flight bout durations measured for flies with expression of WT-CaMKII (THD1;TubGAL80ts>WT-CaMKII) or a constitutively active form of CaMKII, CaMKIIT287D (THD1;TubGAL80ts>CaMKIIT287D), in THD1 marked neurons under various temperature shift conditions as compared to the 18°C condition (n≥20, **p<0.01, Mann-Whitney U-test). (B) Flight bout durations observed with knockdown of CaMKII (CaMKIIRNAi) in dopaminergic neurons (n≥30, *p<0.05, **p<0.01, Mann-Whitney U-test). (C) Locomotor activity of control adult flies and flies with THD1 driven Ala expression (Ala/+ and THD1>Ala; n = 25, p>0.05, ns—not significant; Mann-Whitney U-test). (D) Flight bout durations observed with an Ala control strain under the various temperature shift conditions (Ala/+; n≥20). (E) Quantitative PCR showing normalized mean fold change (± SEM) in TH transcripts under conditions of Ala expression in THD1 neurons, either in pupae or adults (THD1;TubGAL80ts Control and THD1;TubGAL80ts>Ala; n≥3, p>0.05, unpaired t-test). (F) Box plot showing background subtracted fluorescence intensity values of GFP and anti-pCaMKII under various experimental conditions. Cells with GFP intensities ranging from 0–1500 units were chosen for analysis and their distributions were not different in the three conditions (nsyb>mCD8GFP;FMRFaR+; p>0.05, ns—not significant; Mann-Whitney U-test). (G) Box plot showing the ratio of pCaMKII/GFP fluorescence intensity in the three experimental conditions. Peptide stimulation resulted in significantly higher pCaMKII/GFP ratios as compared to addition of the solvent (**p<0.01, Mann-Whitney U-test). (H) Representative images of cells showing endogenous GFP and staining for pCaMKII in the three treatment groups. Scale bar represents 10 μm. (TIF) [file pgen.1007459.s003.tif]

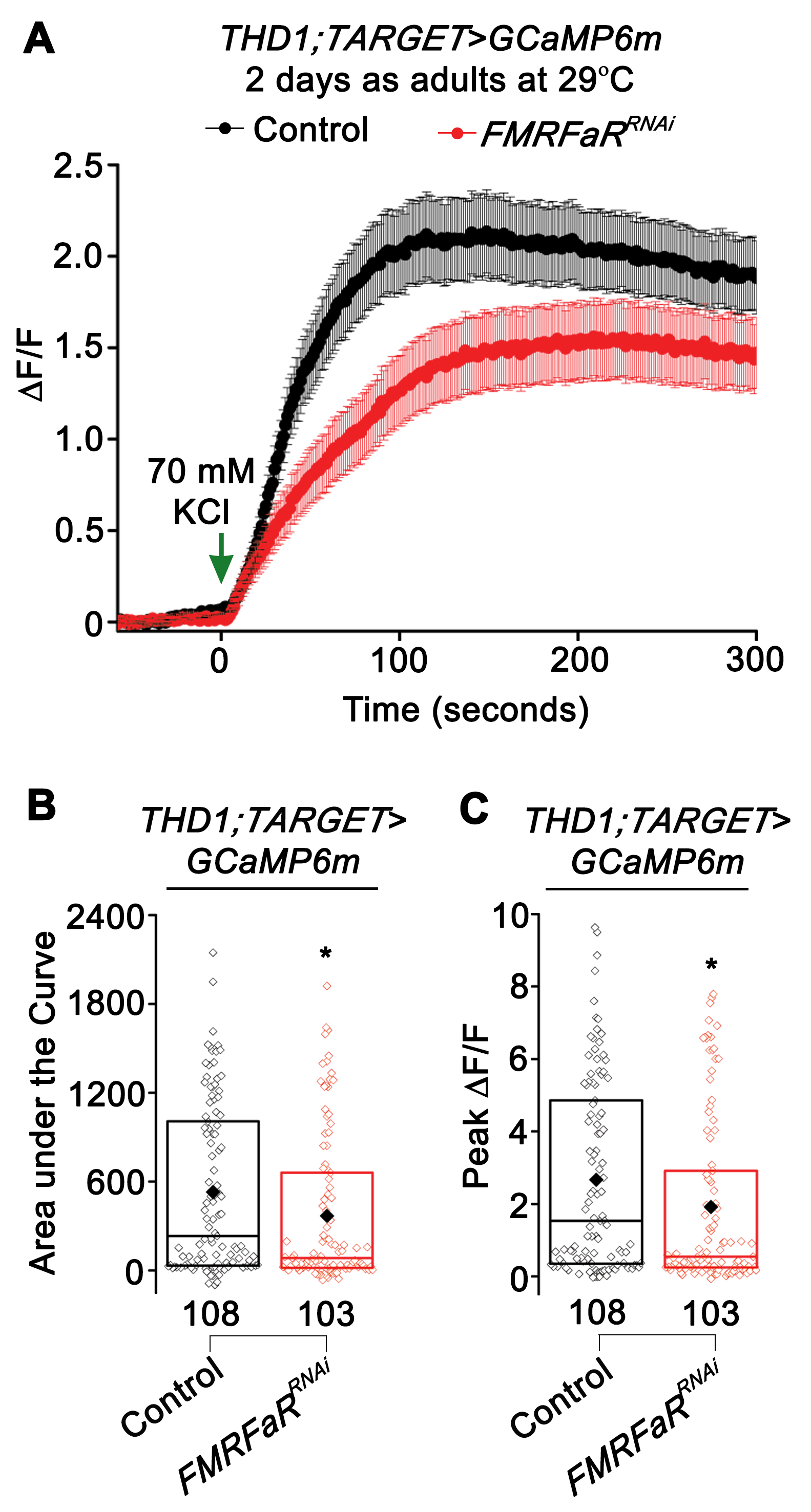

Supplement: S4 Fig — (A) GCaMP6m traces showing normalized mean response (±SEM) of THD1 neurons to KCl in 2 day old adult brains of the indicated genotypes, THD1;TubGAL80ts>GCaMP6m, Control in black; THD1;TubGAL80ts>GCaMP6m;FMRFaRRNAi, in red. (B) Area under the curve and (C) Peak ΔF/F quantified from (A). Numbers below each box plot indicate total number of cells imaged (*p<0.05; Mann-Whitney U-test). (TIF) [file pgen.1007459.s004.tif]

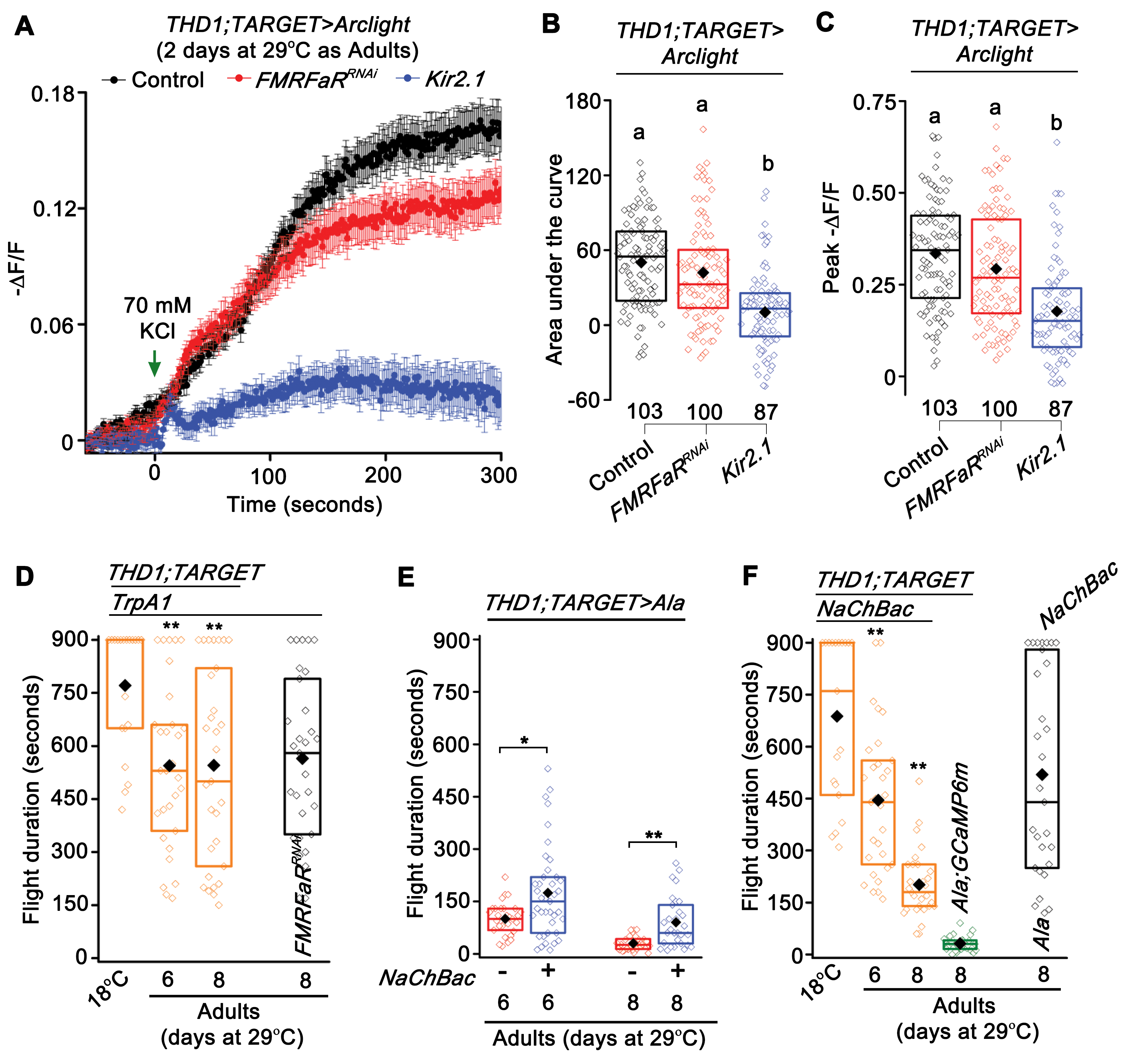

Supplement: S5 Fig — (A) Mean– ΔF/F traces observed in 2 day old adult dopaminergic neurons in response to KCl stimulation, where THD1 neurons express the Arclight transgene in the indicated genotypes, THD1;TubGAL80ts>Arclight, Control in black; THD1;TubGAL80ts>Arclight;FMRFaRRNAi, in red, THD1;TubGAL80ts>Arclight;Kir2.1 in blue. (B) Area under the curve and (C) Peak–(ΔF/F) calculated from (A). Expression of the hyperpolarizing channel, Kir2.1, significantly reduced the response to a depolarizing KCl stimulus. Numbers below each box plot indicate total number of cells imaged (One-way ANOVA followed by post-hoc Tukey’s test; the same alphabet above each bar represents statistically indistinguishable groups; different alphabet represents p<0.01). (D) Flight durations observed for flies expressing just the dTrpA1 transgene in adult THD1 neurons as compared to 18°C control (THD1;TubGAL80ts>dTrpA1; n≥20, **p<0.01, Mann-Whitney U-test). Flight duration of a control genotype used in Fig 6E (dTrpA1/+;FMRFaRRNAi/+—black bar). (E) Rescue of flight deficits observed upon expression of NaChBac, in the background of CaMKII inhibition (Ala) in THD1 neurons in 6 and 8 day old adults (THD1;TubGAL80ts>Ala;NaChBac compared to THD1;TubGAL80ts>Ala; n≥30, *p<0.05, **p<0.01, Mann-Whitney U-test). The rescues are presumably due to increased membrane excitability. (F) Flight bout durations observed with expression of just the NaChBac transgenes in THD1 neurons under control condition (18°C) and for 6 or 8 days as adults (THD1;TubGAL80ts>NaChBac; orange bars; n≥20, **p<0.01, Mann-Whitney U-test). Flight times observed with expression of a GCaMP6m transgene in the background of Ala expression in adult THD1 neurons (THD1;TubGAL80ts>Ala;GCaMP6m; green bar compared to THD1;TubGAL80ts>Ala, 8 days as adults at 29°C shown in Fig 4C are not significantly different; n≥20, p>0.05, Mann-Whitney U-test). Flight duration of a control genotype used in S5E Fig (Ala/+;NaChBac/+—black bar). (TIF) [file pgen.1007459.s005.tif]

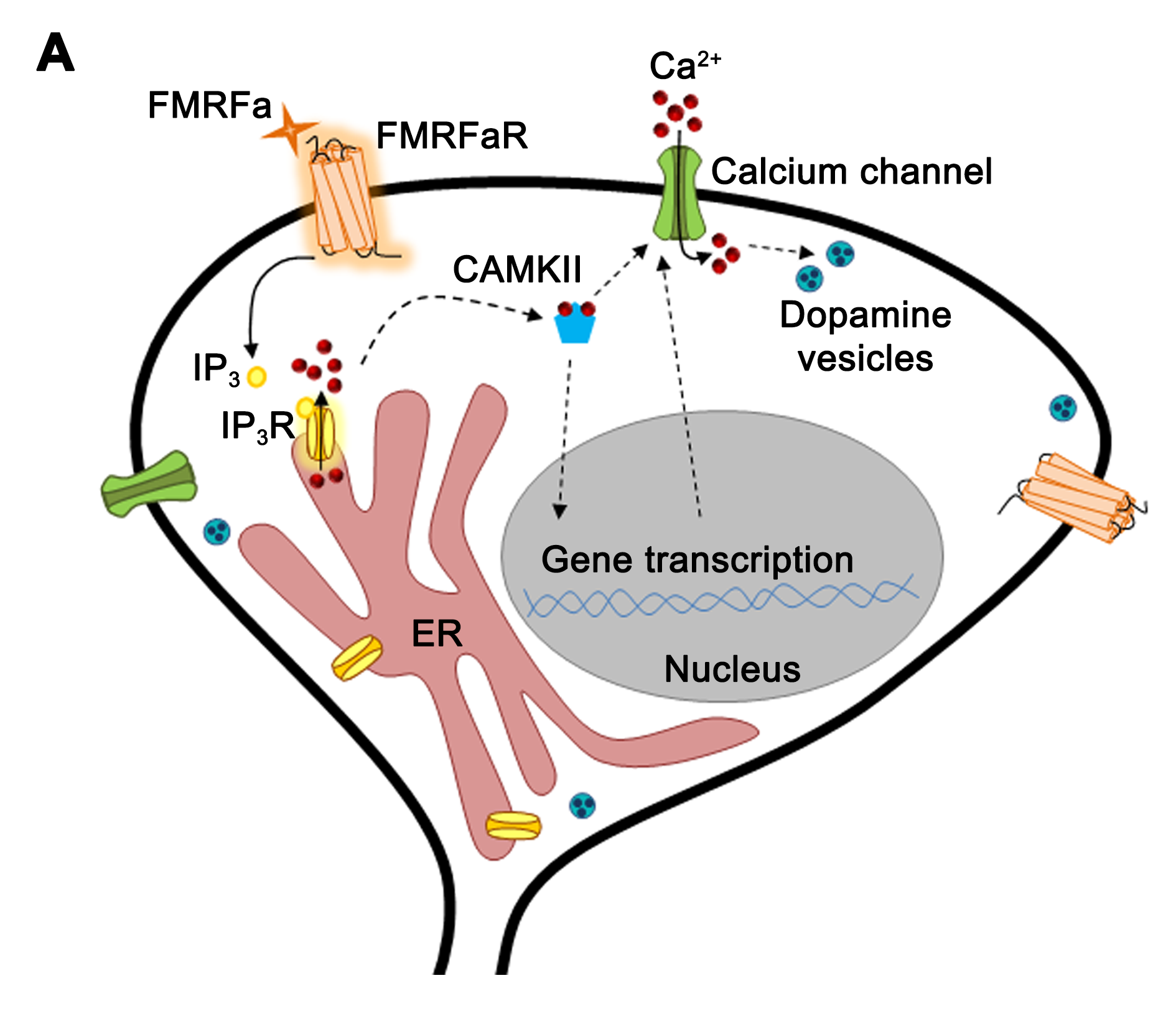

Supplement: S6 Fig — (A) Proposed signaling mechanism in dopaminergic neurons for regulation of flight bout durations by FMRFaR. The model illustrates that FMRFaR stimulation and downstream IP3R-mediated Ca2+ release could possibly activate CaMKII. Our data suggest that the FMRFaR and CaMKII are required in dopaminergic neurons for optimal membrane excitability. The effect on membrane excitability maybe by direct modification of membrane channels or by regulation of their expression levels. We further predict that this excitability is required for exocytosis of dopamine containing synaptic vesicles. Schematics are not drawn to scale. (TIF) [file pgen.1007459.s006.tif]
